# Progress on Gut Health Maintenance and Antibiotic Alternatives in Broiler Chicken Production

**DOI:** 10.3389/fnut.2021.692839

**Published:** 2021-11-17

**Authors:** Qidong Zhu, Peng Sun, Bingkun Zhang, LingLian Kong, Chuanpi Xiao, Zhigang Song

**Affiliations:** ^1^Department of Animal Science, Shandong Agricultural University, Taian, China; ^2^Department of Nutrition Technology, Shandong Hekangyuan Cooperation, Jinan, China; ^3^Department of Animal Science, China Agricultural University, Beijing, China

**Keywords:** gut, health, antibiotic, antibiotic-free strategies, broilers

## Abstract

The perturbation of gut health is a common yet unresolved problem in broiler chicken production. Antibiotics used as growth promoters have remarkably improved the broiler production industry with high feed conversion efficiency and reduced intestinal problems. However, the misuse of antibiotics has also led to the increase in the development of antibiotic resistance and antibiotic residues in the meat. Many countries have enacted laws prohibiting the use of antibiotics in livestock production because of the increasing concerns from the consumers and the public. Consequently, one of the most significant discussions in the poultry industry is currently antibiotic-free livestock production. However, the biggest challenge in animal husbandry globally is the complete removal of antibiotics. The necessity to venture into antibiotic-free production has led researchers to look for alternatives to antibiotics in broiler chicken production. Many strategies can be used to replace the use of antibiotics in broiler farming. In recent years, many studies have been conducted to identify functional feed additives with similar beneficial effects as antibiotic growth promoters. Attention has been focused on prebiotics, probiotics, organic acids, emulsifiers, enzymes, essential oils, tributyrin, and medium-chain fatty acids. In this review, we focused on recent discoveries on gut health maintenance through the use of these functional feed additives as alternatives to antibiotics in the past 10 years to provide novel insights into the design of antibiotic-free feeds.

## Introduction

**Figure 1:**
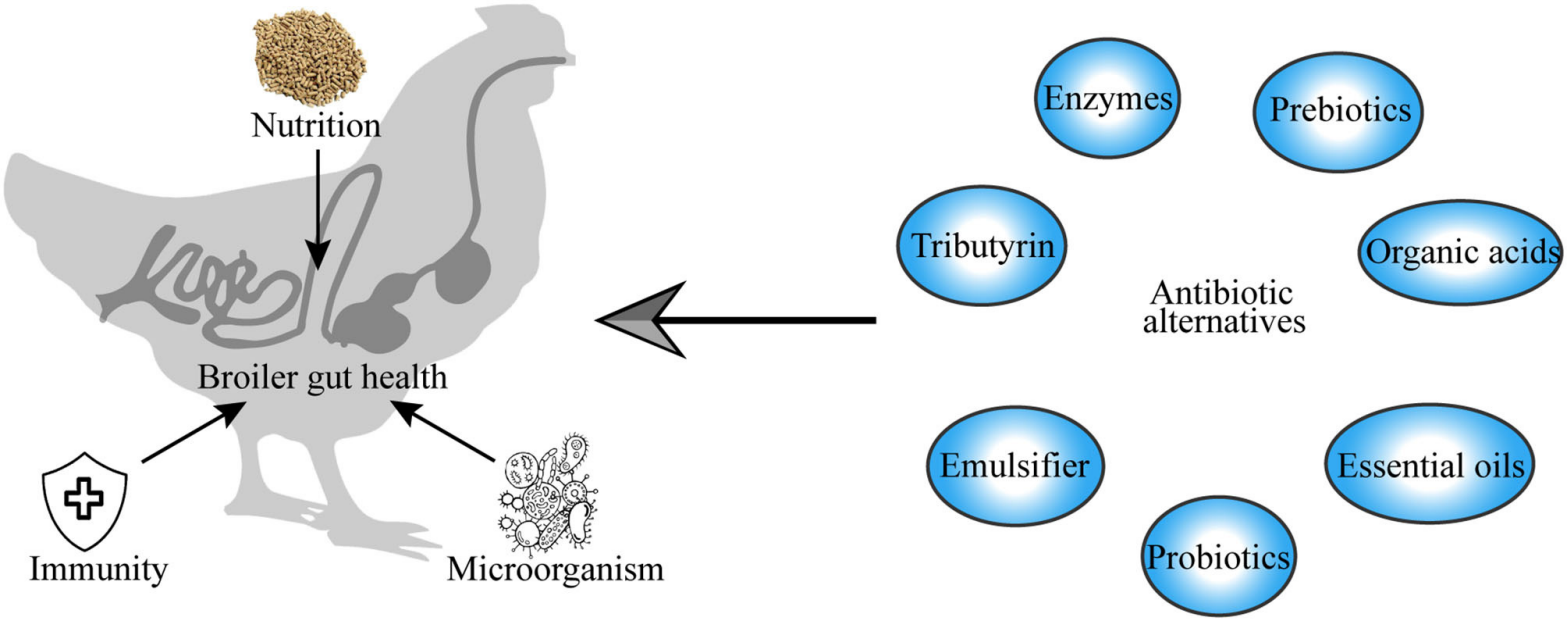
Graphical abstract.

Gut health is an increasingly important topic in broiler chicken production. The rapid rise in the global human population has increased the demand for animal protein for human nutrition, which consequently led to the intensive production of broiler chickens to meet the demand for food, causing unintended gut health problems and performance impairment in broiler chickens. Intestinal diseases are associated with gut mucosal barrier leakage, inflammation, and gut microbiome dysbiosis. For a long time, broiler production has relied on the use of antibiotics, which have led to significant improvements in the growth performance of broiler chicken and have helped in the fight against bacterial infections (1–3). Antibiotics have demonstrated significant value in terms of the enhancement of health and productivity in broiler chickens. However, their misuse in intensive livestock production has led to public and consumer concerns about antibiotic residues in the meat and the development of antibiotic resistance among pathogenic bacteria, with serious implications for human and animal health and the environment. This has led to the enactment of legal regulations prohibiting the use of antibiotics in broiler production to alleviate its risks to human and animal health, as well as threats to the environment (4, 5). This has led to the need to identify alternatives to antibiotics that can be used to fight gut pathogens that cause intestinal diseases (Figure 1).

This review aims to identify the causes of gut health problems, show the reported results of various regulatory measures or alternative antibiotics, and analyze the feasibility of feeding without antibiotics.

## Lessons From the Gut Health Impairment in Broilers

**Figure 2:**
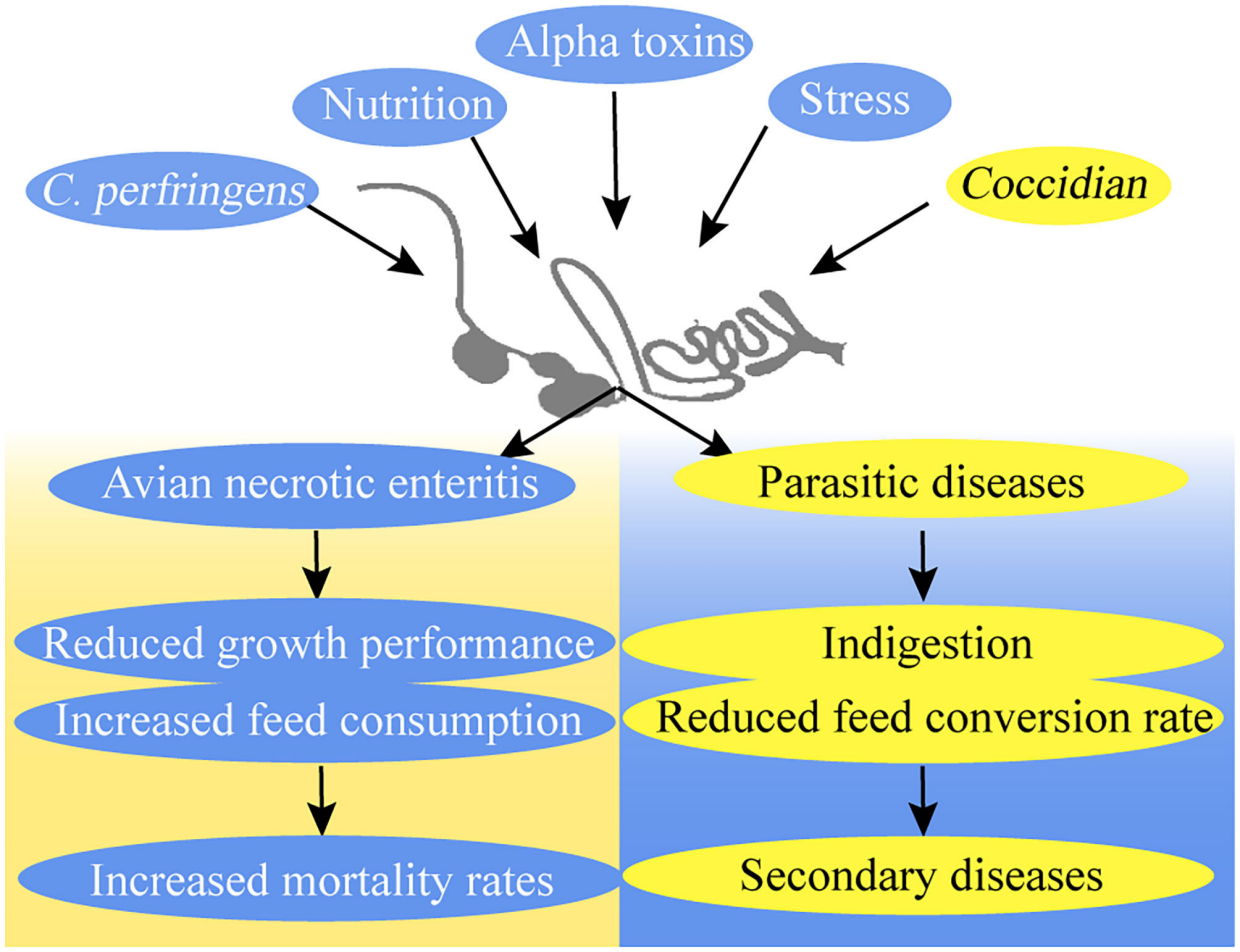
Avian necrotic enteritis and parasitic diseases in broilers.

Poultry production losses caused by avian necrotic enteritis (NE) and parasitic diseases, such as coccidiosis, have become a global challenge for the poultry industry (6, 7). Since the ban on antibiotics in animal feed, the high prevalence of NE and coccidiosis has become a major cause of mortality in broilers (8–10) (Figure 2).

Necrotic enteritis increases feed consumption and mortality rates but reduces the growth performance of broiler chickens (11). Several reasons can be cited as to why broilers contract NE. Nutrition, stress, and coccidiosis are predisposing factors that influence the incidence of NE (8). *Clostridium perfringens* is a crucial factor for the development of NE in broiler chickens because of its negative influence on the epithelial barrier (12, 13). *C. perfringens* uses and releases more than 16 toxins that cause histotoxic and intestinal infections in animals. Different toxins may bring virulence flexibility to *C. perfringens*, thereby causing a series of diseases (14). C. *perfringens* is also one of the most common contaminants in feeds (15). Alpha toxins are *C. perfringens* type A product, which causes gas gangrene (16, 17). Early studies on NE showed that alpha-toxin is the main virulence factor for the development of the disease, but recent studies proposed that alpha-toxin is not an essential virulence factor in the pathogenesis of NE in poultry (8, 13, 18–20). NetB is identified in a *C. perfringens* strain isolated from NE in broilers and has considerable potential for novel vaccines against NE in broilers (19, 21–23).

Coccidiosis is a recurring disease that endangers the intestinal health of broilers and causes economic losses in the chicken industry (24). The effects of coccidiosis include indigestion and increased feed conversion rate, weight gain, and susceptibility to secondary diseases in infected broilers (25). The main cause of coccidiosis outbreaks is the protozoan *Eimeria* species, and the infection route is through fecal–oral transmission (2, 9). The *Eimeria* species increase their environmental survival through their ability as oocysts and their drug resistance (9, 10). *Eimeria* induces plasma protein leakage by damaging epithelial cells in the intracellular phase, which includes mucus production enhancement and the secretion of collagenases and collagenolytic enzymes in the intestines (18, 20).

## Impairment Factors of Gut Health in Broilers

**Figure 3:**
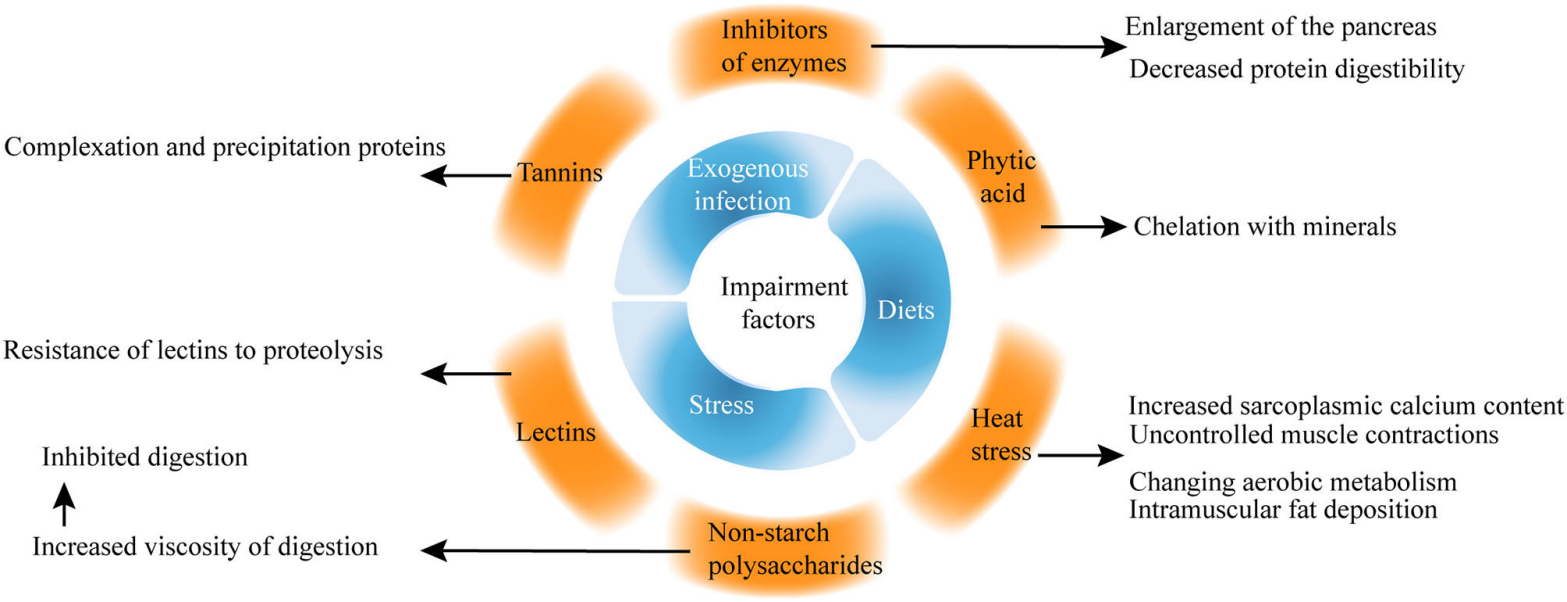
Impairment factors of gut health in broilers.

Among the wide range of significant factors affecting broiler health, stress, diets, exogenous infection, and water are the most common indicators. Recently, more studies on the impairment factors of the intestinal health of broilers have focused on phytic acid, non-starch polysaccharides (NSPs), inhibitors of enzymes, lectins, and heat stress (Figure 3).

### Phytic Acid

Phytic acid is a natural antioxidant found naturally in the form of salts and is present in cereals, vegetables, nuts, and natural oils (26). For example, phytic acid forms insoluble salts with minerals, including phosphorus, calcium, zinc, magnesium, and copper. Phytic acid increases the mucin (MUC) excretion and endogenous nutrient losses, which are hazardous to intestinal health.

Early studies showed that phytic acid is considered an antinutritional component because of its ability to chelate with minerals, but recent studies proposed that phytic acid performs well in various pathological conditions, intoxication, and cancer (27, 28). Phytic acid protects the integrity of the cytoplasmic membrane of intestinal cells against the harmful effects of deoxynivalenol, which is related to NE (29).

Low-phytate pea affects iron bioavailability, physiological status, gut microbiota composition and metagenome, and intestinal function (30). Phytase optimizes the phosphate transporter gene expression and improves efficient dietary phosphorus utilization (31).

### Non-Starch Polysaccharides

Non-starch polysaccharides, together with resistant starch and lignin from the dietary fiber, are found in plants especially in the endospermic cell wall of multiple kinds of seeds (32). Many viscous NSPs are present in the diet of chickens, leading to increased fermentation in the small intestine, which is harmful to the performance and gut health of poultry.

In the past, NSPs are considered an antinutritional factor because they increase the viscosity of digests and inhibit digestion. However, the beneficial effects of NSPs cannot be denied. NSPs can promote the immune system, reduce inflammation (33–37), and modulate the gut microbiota (38).

### Inhibitors of Enzymes

The inhibitors of enzymes, including trypsin, chymotrypsin, carboxypeptidases, elastase, and α-amylase, are important naturally occurring antinutritional factors. Soybeans are a major source of trypsin inhibitors among food and feed products (39). Trypsin inhibitors cause the enlargement of the pancreas and decrease protein digestibility.

Toasted soya beans decrease the trypsin inhibitor activity. Nontoasted full-fat soya beans increase subclinical NE lesions in the gut compared with toasted full-fat soya beans (40). The high content of trypsin inhibitors negatively affects nutrient utilization in the diets of broiler chickens (41).

### Lectins

Lectins, which can be subdivided into hololectins, merolectins, chimerolectins, and superlens, are widely distributed in all plant tissues.

Plant lectins have oral toxicity to higher animals because of the resistance of lectins to proteolysis (42). Tannins are naturally occurring water-soluble polyphenolic compounds with the ability to complex and precipitate proteins in aqueous solutions and are responsible for the astringent taste of many fruits and vegetables (43).

### Heat Stress

Heat stress can adversely affect welfare and productivity by altering the activity of the neuroendocrine system of broilers (44). Heat stress induces perturbation in the gut microbiome of chicken (45, 46) and impairs nutrient transport and gut health by modulating oxidative stress and inflammation (47).

## Antibiotic-Free Management Strategies in Broilers

**Figure 4:**
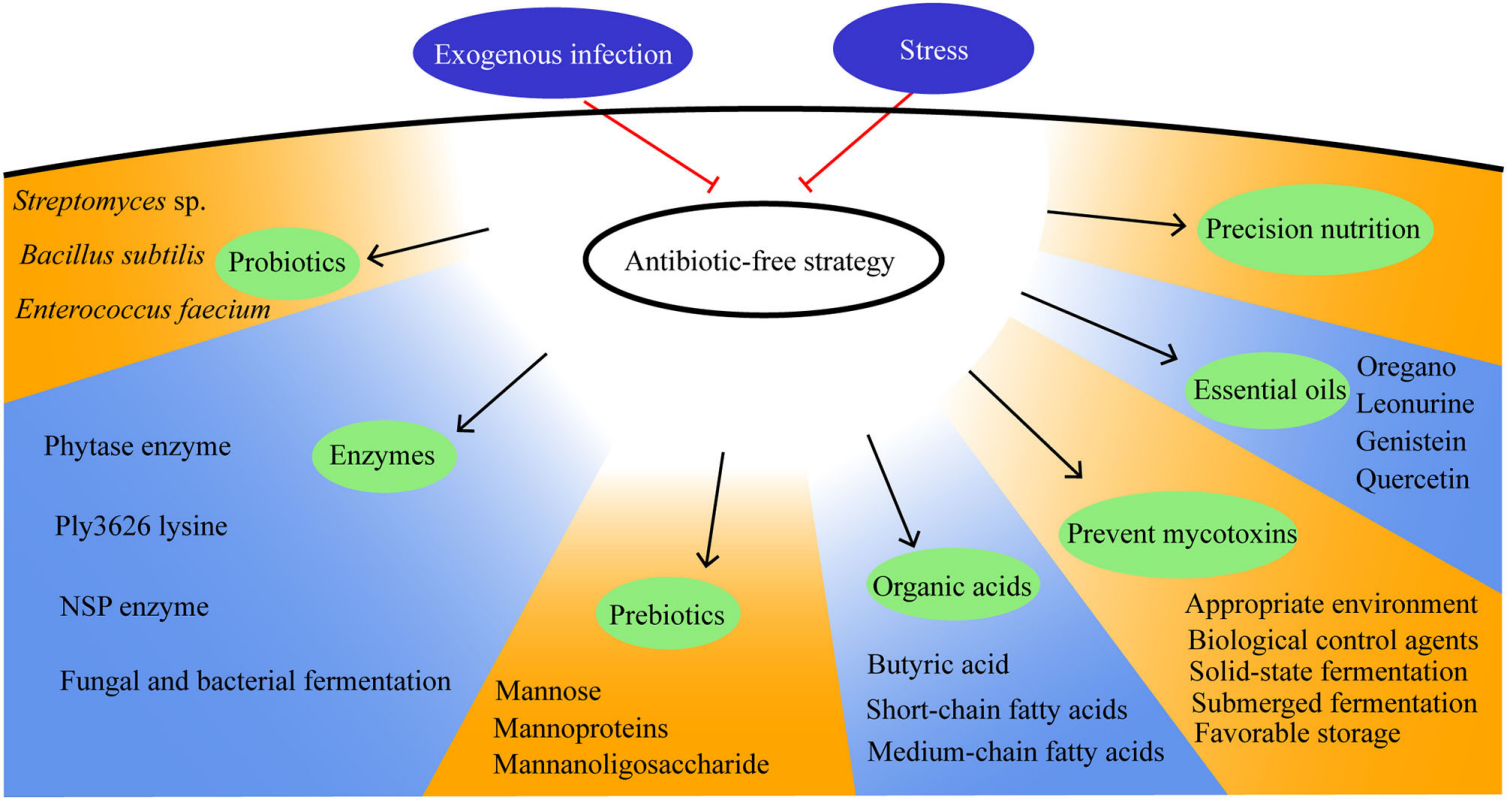
Antibiotic-free management strategies in broilers.

Housing conditions, pathogen exposure, and dietary nutrients play major roles in moderating the gut health of broilers. Therefore, improving the gut health includes reducing stress, promoting precision nutrition, preventing exogenous infection, and having concern over how antibiotic-free management strategies are used and how the breeding environment can be improved. In this study, a review of antibiotic-free management strategies will be detailed and will be discussed from the feed quality control, feed additive enzymes, prebiotics, probiotics, organic acids, and plant extract (Figure 4).

### Feed Quality Control

Mycotoxins, including aflatoxins, ochratoxin A, fumonisins, trichothecenes, zearalenone, emerging *Fusarium* mycotoxins, ergot alkaloids, and patulin, are the secondary metabolites produced by fungi and can cause mycotoxicosis (48). The strategies for its prevention include the use of biological control agents, appropriate environmental factors, and favorable storage practices (49–52). Natural means, such as thermal insulation, radiation treatment, and low-temperature plasma; chemical methods, such as oxidation, reduction, hydrolysis, alcoholysis, and absorption; biological methods with the use of biological agents can eradicate mycotoxins (53). Prevention is the most important strategy in the fight against mycotoxins.

Fermentation is a new cheap way to improve the nutritional value of feed ingredients for broilers. Fermented poultry feeds use solid-state (SSF) and submerged (SmF) fermentation methods (54). Phytase is produced from fungal strains during SmF. Fermented feeds affect the growth performance, gastrointestinal tract microecology, gut morphology, immune system, and welfare of poultry.

Fermented feeds can improve growth performance, immune function, and antioxidant capacity in chickens. Fermented-feed diets for chickens reduce the antinutritional factors process that modulates host T-cell proliferation, T helper type 1, and T helper type 2 cytokine production; many antioxidation are associated with nuclear factor-κB (NF-κB) activation (55).

### Feed Additive Enzymes

Fungal and bacterial fermentation methods produce feed additive enzymes that maximize feed conversion efficiency. Enzymes facilitate component, protein, phytate, and glucan degradation and improve diet digestion. The phytase (500 FTU per kg) enzyme increases the villus width and decreases the crypt depth, which improves the average daily gain (ADG) (56). Ply3626 lysine (enzyme) may be exploited as an antibacterial for the treatment of *C. perfringens* infection and is proposed as a biocontrol agent in poultry production (57). Feed additive enzymes ameliorate the deleterious effects of coccidiosis on the gut health and function of broiler chickens (58).

Phytase (500 and 1,500 units per kg) supplements improve phosphorous availability and have high plasma concentrations of kynurenine and creatinine, and low concentrations of histamine and cis-4-hydroxyproline (59). The NSP enzyme (150 mg per kg) affects the digestive function, serum cholecystokinin, pancreatic lipase, and amylase enzyme activities, and mRNA expression in broilers (60). Exogenous multienzyme complexes increase the intestinal peptide transporter 1, facilitative glucose transporter (GLUTs; e.g., GLUT2), acetyl-CoA carboxylase, and interleukin (IL)-2 expression levels, which improve the absorption of micronutrients and enhance the growth performance of broiler chickens (61).

### Prebiotics

Prebiotics stimulate the growth and activity of bacteria in the intestines through their fermentable properties, benefitting the health of broilers. Prebiotics are composed of short-chain polysaccharides and oligosaccharides. Prebiotics cannot be digested by broilers but can be metabolized by gut microbes to produce short-chain fatty acids (62). Prebiotics have been shown to reduce *Campylobacter* relative to its abundance in cecal contents and other intestinal sections of the gut. Prebiotics, such as propionic, acetic, and butyric acids, have a positive effect on the performance of broilers, contribute to their gut health, and can be a good alternative to antibiotics. Prebiotics change the composition of cecal microbes in the gut, leading to changes in the Proteobacteria and the genus and family of bacteria and improving the performance of broilers (56).

The addition of a product rich in mannose, mannoproteins, and mannanoligosaccharide (0.2 and.5%) in feeds significantly increases the number of intestinal villus cells, and mannanoligosaccharide confers gut health benefits over antibiotics through the reduction of pathogenic bacteria, morphological development, and increased colonization by beneficial bacteria (63, 64). The ingestion of *in ovo* prebiotics in the chicken embryo is an effective practice and alternative to antibiotic growth promoters in broilers (65).

Curcumin (50 and 100 mg per kg) supplementation induces the expression of nuclear factor E2-related factor 2 (Nrf2) and Nrf2-mediated phase II detoxifying enzyme genes and increases the glutathione content and glutathione-related enzyme activities (66). Yeast cell wall supplementation modulates the intestinal glutathione pathway, proteolytic enzyme activity, nutrient transport, and messenger RNA (mRNA) expression levels of neutral, cationic, and oligopeptide transporters (67). Nanoparticles of Fe_3_O_4_ (50 mg per kg) prevent the invasion of *Salmonella enteritidis* through the regulation of phosphatidylinositol-3-kinase (PI3K)/protein kinase (Akt)/mammalian target of rapamycin signaling pathways (68, 69).

### Probiotics

Probiotics, as a live microorganism feed supplementation, improve growth, feed efficiency, and intestinal health (70). *Enterococcus faecium* (5 ×108 or 5 × 109 cfu per kg feed), *Streptomyces* sp., and *Bacillus subtilis* (5 × 108 cfu/kg feed) have antibacterial effects on the bacterial microflora in the small intestine. Beneficial microflora contributes to the development of the intestinal immune system and helps in the activation of innate and adaptive immune responses (71, 72).

In terms of coccidiosis, probiotics may also have an anticoccidial role. A study found a protecting effect in probiotic preparations that help reduce the negative effects of coccidiosis (70). Probiotics help minimize the risk of coccidiosis spread and maintain gut health.

Probiotics stimulate endogenous enzymes, reduce metabolic reactions, and promote vitamin or antimicrobial substance production. Bacteriocins are antimicrobial peptides of bacterial origin and have antimicrobial activities that inhibit the production of toxins and the adhesion of pathogens (73).

Probiotics induce IFN-γ, MUC2, transforming growth factor-beta 4 cytokine expression patterns, and the relative abundance of specific bacterial taxon changes in the cecal microbiota (74). Virginiamycin supplementation enhances the epithelial barrier integrity and increases the expression levels of IL-2 and INF-γ, and *B. subtilis* supplementation improves the growth performance, intestinal immunity, and epithelial barrier integrity. The expression levels of IL-2 and INF-γ are downregulated (75). *E. faecium* supplementation upregulates the expression of intestinal-type IIb sodium-dependent phosphate cotransporter mRNA, increases the concentration of serum alkaline phosphatase, changes the gut microbiota populations, and increases the utilization of phosphorus (76). *Bacillus subtilis* BYS2 supplementation improves the production performance, immunity, and disease resistance; promotes innate immune response, increases the expression levels of interferon (IFN)-stimulated genes and β-defensins, and upregulates inflammatory cytokines (77). *Lactobacillus* supplementation increases the expression of sugar transporter genes (e.g., GLUT2, GLUT5, sodium-glucose cotransporter [SGLT] 1, and SGLT4) and improves the bacterial population of cecal contents (78).

### Organic Acids

Short-chain fatty acids, medium-chain fatty acids, and other organic acids are used as an alternative to antibiotics to reduce the pathogenic bacteria in the gut based on their antimicrobial activity outside the gut. The antibacterial effect of organic acids is specific to species (79). The addition of organic acids causes a decrease in *Escherichia coli, Salmonella, Campylobacter*, and other potentially pathogenic bacteria, which result in a beneficial effect on the gut health of broilers (80).

Growth, feed conversion rate, and feed utilization can be promoted by adding organic acids (0.06% Galliacid, 0.1% Biacid, or 0.02% Eneramycin) to the feed or drinking water at appropriate times (81). Butyric acid improves the growth performance of feed proteins of low digestible sources in chickens (82). Butyric acid is an energy source of Intestinal epithelial cells (IECs) that stimulate proliferation and differentiation. Thus, improving the feed efficiency of diet supplementation with organic acids (formic, propionic, and acetic acid) can positively affect the cecal microbiota composition and ileal microbial glycolytic enzyme activity (83). L-theanine is available as a dietary supplement, used as the best natural feed additive, and can improve the growth performance, immunity, intestinal morphology, and antioxidant status of chickens (84).

Taurine supplementation alleviates fat synthesis by suppressing the liver X receptor alpha pathway and decreasing lipid accumulation in the liver (85). Glutamine inducing the Nrf2–Keap1 pathway modulates the muscle glutamine level and improves the resistance of heat-stressed broiler muscles to oxidative damage (86). Diets supplemented with a blend of organic acids prime the immune cells, and boost the immune system of chicks. Heterophils have high expression levels of IL10, IL1β, and C-X-C Motif Chemokine Ligand 8 mRNA (87). Taurine improves immunity by regulating the PI3K–Akt signaling pathway (88).

### Plant Extract

Plant extracts are phytogenic feed additives that can be divided into phenolics, nitrogen-containing alkaloids, sulfur-containing compounds, and terpenoids based on their biosynthetic origin. Ginger and oregano are suitable for poultry feed rather than garlic and rosemary because they appear to be less sensitive to odor (89, 90). Similarly, essential oils are promising alternatives to growth promoter antibiotics. Essential oils can play preventive and curative roles in NE in broilers (91). Adding oregano essential oil (300 and 600 mg per kg) in broiler chicken feed increases the ADG (92). Guduchi (*T. cordifolia*) has a positive effect on poultry growth performance, enhances the immune function in birds, and is used as a potent immunomodulator and an active antimicrobial agent in poultry (93).

Essential oils interfere with the modulation of immune responses and inflammation (5, 94). The antibacterial effects of essential oils disrupt the structure of the membrane and inner cell structures *via* their lipophilic characteristics and related ability to penetrate through the cell wall and cytoplasmic membrane (95). The antioxidant effects of essential oils are observed to be connected to the reduction of tumor cell proliferation either by apoptosis or necrotic effects (96). Ginger oil and carvacrol can influence the digestibility and speed of feed passage through the digestive tract, increase the secretion of saliva, bile, and mucus, and enhance enzyme activity. Essential oils with saponins can promote the growth performance of broilers and increase the protein digestibility and absorption of dietary nutrients that are related to intestinal development and protease activity (97).

Grape seed proanthocyanidin ameliorates aflatoxin B_1_-induced immunotoxicity and oxidative damage by modulating the NF-κB and activating the Nrf2 signaling pathways (98). Leonurine hydrochloride supplementation improves intestinal mucosal disruption by regulating the expression of tight junction (TJ) proteins and inhibiting the activation of the NF-κB/mitogen-activated protein kinase (MAPK) signaling pathway (99). Genistein ameliorates the growth performance of chicks with intestinal injury and prevents the Lipopolysaccharides (LPS)-induced NF-κB-dependent cytokine and MAPK cascade signaling (100). Epigallocatechin-3-gallate increases the antioxidant activity, regulates the MAPK/Nrf2 signaling pathway, and upregulates the P-38MAPK, Nrf2, and heme oxygenase 1 expression levels (101). Quercetin supplementation decreases the expression of NF-κB inhibitor-alpha mRNA and increases the expression levels of TNF-α, TNF receptor-associated factor-2, TNF receptor superfamily member 1B, NF-κB p65 subunit, and IFN-γ mRNA, thereby improving the immune function *via* the NF-κB signaling pathway triggered by TNF-α (102).

## Conclusion

The livestock industry has paid considerable attention to the issues of antibiotics, and topics on intestinal health have become the spotlight. The purpose of antibiotic alternatives is to keep the environment and consumers healthy and maintain low mortality and high animal production. Identifying a single “ideal” solution within the wealth of options for gut health control is difficult. Several measures and alternatives to antibiotics can be used in conjunction with one another to achieve the perfect gut health.

In recent years, feed additive enzymes, prebiotics, probiotics,organic acids, and plant extracts play an increasingly important role in fighting infectious diseases and stimulating poultry growth. The use of antibiotic alternatives has addressed the problem of antibiotic resistance and residues in the food and environment, which can promote gut health. In addition, this strategy maintains favorable sanitary conditions and ensures high-quality feed ingredients. Precise nutrition is also critical.

## Author Contributions

QZ wrote the original manuscript and contributed much to the revised figures. PS wrote the manuscript and revised it critically for important intellectual content. BZ edited the manuscript. LK and CX drawed the original figures. ZS designed the profiles of the manuscript. All authors contributed to the article and approved the submitted version.

## Funding

This work was supported by the Natural Science Foundation of Shandong Province (ZR2020MC170), the National Key R&D Program of China (2018YFD0501401-3), and the Shandong Province Agricultural Industry Technology (SDAIT-11-08).

## Conflict of Interest

The authors declare that the research was conducted in the absence of any commercial or financial relationships that could be construed as a potential conflict of interest.

## Publisher's Note

All claims expressed in this article are solely those of the authors and do not necessarily represent those of their affiliated organizations, or those of the publisher, the editors and the reviewers. Any product that may be evaluated in this article, or claim that may be made by its manufacturer, is not guaranteed or endorsed by the publisher.
